# Public opinion trends in American society: Lessons from social science infrastructure

**DOI:** 10.1093/pnasnexus/pgag002

**Published:** 2026-02-24

**Authors:** James N Druckman, Alice Brocheux, Pauline Gordula, Hope E Marsh, Dot Sawler, Daniel Sun, Yi-Fan William Zhu

**Affiliations:** Department of Political Science, University of Rochester, 333 Harkness Hall, Rochester, NY 14627-0146, USA; Department of Political Science, University of Rochester, 333 Harkness Hall, Rochester, NY 14627-0146, USA; Department of Political Science, University of Rochester, 333 Harkness Hall, Rochester, NY 14627-0146, USA; Department of Political Science, University of Rochester, 333 Harkness Hall, Rochester, NY 14627-0146, USA; Department of Political Science, University of Rochester, 333 Harkness Hall, Rochester, NY 14627-0146, USA; Department of Political Science, University of Rochester, 333 Harkness Hall, Rochester, NY 14627-0146, USA; Department of Political Science, University of Rochester, 333 Harkness Hall, Rochester, NY 14627-0146, USA

**Keywords:** General Social Survey, American National Election Studies, affective polarization, trust, happiness

## Abstract

How have individual and national welfare evolved over time in the United States? We use long-standing National Science Foundation–supported surveys—the American National Election Studies and the General Social Survey—to address this question. Our selected examples suggest measures of individual welfare (economic, health, happiness) have remained relatively stable, although with some recent acute declines (e.g. since 2020). More dramatically, beliefs about national welfare such as satisfaction with how democracy works, affective polarization, political efficacy, and confidence in various institutions have moved in negative directions over decades. These trends paint a picture of a stressed nation. They additionally highlight how descriptive survey data can be used to understand and raise questions about American society. Public opinion trends play a vital role in the social scientific process and these data are only available due to generational investments in gold-standard time series survey data.

There is an eternal aspiration that society today outperforms society in the past. Of course, this never comes to fruition as key economic, social, and political trends ebb and flow even in “good times.” The ostensible zeitgeist in contemporary America is that times are not good—divisions are high, moods are low, and trust has frayed. The point of this perspective article is to descriptively present public opinion trends about individual and national welfare in the United States. In doing so, we make 2 points relevant to the social sciences.

First, the selected trends provide a portrait of the country's trajectory over decades. They show general stability along with some recent acute declines in individuals’ economic satisfaction, health, and happiness. National welfare trends reveal large, virtually monotonic, declines in satisfaction with democracy, political efficacy, and confidence in institutions, as well as an enormous increase in affective polarization. Subgroup analyses reveal emergent partisan gaps in institutional confidence: for the most part, 50 years ago, partisans’ opinions differed only when it came to confidence in labor and business, but now they diverge with regard to many institutions (e.g. education, science, organized religion).

Second, these and other trends are only available due to a national commitment to track public opinion over time via infrastructure projects supported by the National Science Foundation (NSF). More specifically, the data come from the American National Election Studies (ANES) and the General Social Survey (GSS), 2 of the 3 long-term time series survey projects supported by the NSF's Research Infrastructure in the Social and Behavioral Sciences Program. (The other survey is the Panel Study of Income Dynamics.)

The ANES is a study of American elections that began in 1948 and uses probability samples (1). The GSS monitors social characteristics and attitudes in the United States; it launched in 1972 and fully adopted probability methods by 1977 (2). The ANES and GSS provide unmatched information on societal dynamics over time (3–5). Yet, their use of probability samples comes with sizeable expense. We consequently start in the next section with a discussion of survey methodology to establish the credibility of these data and to be transparent about investment tradeoffs which we return to in the conclusion.

## Survey sampling

The study of public opinion has a long history, extending back hundreds of years, if not more (6). An inflection point came in the 1930s with the advent of probability samples (7). A probability sample is one where the researcher selects respondents from a frame that reflects the entire population (e.g. all US residents), and where each respondent in the population has a known, nonzero probability of being included. This if often done with random selection. In theory, a probability sample offers a bias-free estimate of a population value (e.g. how efficacious people feel in influencing what government does) with measurable errors. Probability samples are a remarkable innovation insofar as they allow researchers to make a claim about a large group (e.g. all residents) by surveying a small number of group members, such as 1,500 out of 340,000,000.

Of course, nothing so powerful is easy. Indeed, collecting a national probability sample is costly and time-consuming. It requires identifying the entire population, in our example that would mean 340,000,000 residents in the United States. It then entails drawing the sample with a random mechanism (or an approach akin to a random mechanism) to identify the 1,500 residents and enticing them to participate in a survey. Several practical challenges exist, with a notable difficulty being garnering responses.

The response rate can roughly be thought of as the proportion of people who complete the survey out of the number of people asked to do so. In the early days of surveys, response rates were high, but over time they have dramatically dropped. For instance, in 1976, 2000, and 2024 the response rates for the GSS were 75%, 70%, and 45%, respectively. The analogous response rates for the ANES were 70%, 61%, and 33%, respectively. (Note that the 1976 and 2000 surveys were face-to-face. The 2024 GSS randomly assigned respondents to first take the survey on-line or in-person, followed by the other mode if a respondent did not comply. The 2024 ANES response rate listed is for their face-to-face survey. Their Web survey yielded a response rate of 38%. The GSS and ANES use slightly different computations for their response rates and thus should not be directly compared with one another.) Other probability surveys, particularly those done via phone, have much lower response rates (e.g. average under 10%) (8 , 9). Returning to our example, if only 33% of the 1,500 contacted respond, the result is an actual sample of 495; if one wanted 1,500 actual respondents, they would have to draw an initial sampling frame of 4,546 individuals that they then approach.

The decline of response rates raises the question of whether probability samples still warrant the investment. Low response rates, though, are not fatal because they are technically orthogonal to biased estimation (10–12). For instance, returning to our earlier example, if the 67% nonrespondents do not differ at all from the 33% respondents, there is no problem in estimating the variable of interest (e.g. political efficacy). What matters is whether the nonrespondents (i.e. unit nonresponse) introduce systematic bias, which occurs when the group of people who choose not to respond differs from the group who choose to respond (e.g. the nonrespondents have less political efficacy than the respondents).

That said, systematic bias due to nonresponse is clearly a concern in some domains. For instance, the polling industry has had well-documented challenges in forecasting vote choice since at least 2016 [(13–16); cf. (7)]. The ANES, for instance, mispredicted the presidential vote in 2024 by roughly 5.7% points (17). Generally, nonprobability samples—in which participants enter the survey in a nonrandom fashion with unknown probabilities, in response to a solicitation—performed as well or better than probability samples in forecasting the presidential vote (17). Moreover, Tyler et al. (18) showed that that recent ANES data are missing not at random, which introduces biases.

One culprit for misestimation is that Trump supporters are systematically less likely to accept an invitation to participate in probability surveys relative to non–Trump supporters. And, poststratification sample weights (e.g. increasing the influence of lower-educated White male sample respondents relative to others in estimating vote choice) have been insufficient to correct for the nonresponse bias (14 ). It is not entirely clear why nonprobability surveys fared better, although it may be that professional respondents common in such surveys are (relatively) more likely to be Trump supporters.

The result is incorrect inferences due to systematic unit nonresponse (19), and the disenfranchisement of these (nonrespondent) opinions in the surveys (20). Whether this is problematic for the ANES remains unclear, as the project does not aim to forecast the vote, a particularly difficult task [cf. (21)]. Its mission instead is to advance understanding of election related behaviors and attitudes. The ANES does not even release data in advance of the election for forecasting since that is not their goal. Regardless, we will not resolve debates about forecasting here; instead, we make 4 points relevant to assessing samples.

First, evaluating the appropriateness of a given sample requires attention to its “fit for purpose”—that is, its usage and the inferential goal (22–24). For example, descriptively estimating the values of a variable in a population (e.g. the amount of efficacy among Americans), or the relationship between 2 variables (e.g. between gender and efficacy among Americans), differs in purpose from evaluating if an experimental treatment significantly affects an outcome (e.g. whether receiving a message from an elected official increases efficacy). In the experimental (causal) situation, nonprobability samples have been shown to often replicate probability samples at least in terms of the direction of the relationship (e.g. between the message and efficacy) or what is called sign generalization (25). That is much less the case when it comes to descriptively estimating the values of variables in a population (e.g. efficacy) or the size of associations between two variables (e.g. gender and efficacy). Descriptive goals align more with what the infrastructure projects provide.

Second, in these cases, probability samples ostensibly have empirical advantages. A robust literature suggests that probability samples typically arrive at more accurate estimates of single variables and relationships between variables than nonprobability samples (26–32). That said, nonresponse bias can undermine an inference when the source of nonresponse correlates with the variable of interest (33, 34). As stated, this likely explains the aforementioned misestimates of vote choice. For example, it could be that nonrespondents have systematically lower institutional trust and such individuals disproportionally support Trump (35, 36).

Third, abandoning probability samples has the upside of substantially reducing costs. Yet, it introduces the problem of selection bias of who opts into the survey since nonprobability samples do not have known sampling frames (i.e. addressing the nonresponse problem becomes difficult or impossible) (37). Moreover, there are additional uncertainties that make reliance only on typical nonprobability samples akin to “flying blind” when it comes to population estimates of variables and relationships (11).

Consider that the common alternative is an opt-in nonprobability panel that participants join to take multiple surveys over time (38). These usually draw samples in 2 steps—they recruit people to join the panel (which is different than a time-series panel data) and then they sample from that panel (23). They usually do the latter using quotas that align with population benchmarks (e.g. 50% men and 50% women). The well-known problem here is that one cannot know whether they have chosen all the relevant quotas for the outcomes of interest. For instance, a gender quota would be vital estimating how much confidence Americans have in understanding politics but much less so for voting turning out; there is a gender gap in Americans’ confidence but not in voting turnout (39, 40). While the inclusion of a gender quota may be easy, there is another issue of whether interlocking quotas should be used to ensure, for instance, an accurate distribution of low- and high-income women. The challenge becomes more complicated when there are many outcome variables of interest in the survey such as efficacy, trust, economic evaluation, and so on. For each variable, different sample characteristics may matter. With a probability sample—putting aside nonresponse and related biases—one can assume that measured and unmeasured aspects of the sample reflect the population. Moreover, the total survey error captures the main sources of error (41). It consists of sampling error—that is, the inherent uncertainty from sampling—and nonsampling errors that result from the design of the survey. The nonsampling errors include specification error (i.e. mismeasuring concepts a la face validity), frame error (e.g. excluding relevant parts of the population from being eligible from the sample), nonresponse error, measurement error, and processing error. Put another way, with probability samples, the nonresponse problem can be severe, but it is defined whereas with nonprobability samples, it is difficult to evaluate sampling biases given the lack of an established data generating process (a nonprobability quota sample also is constrained to use quotas only on variables for which clear population values are available).

Finally, probability samples are generally better equipped to address emergent concerns about response validity. Opt-in nonprobability samples that collect data on-line do not know the identity of their respondents, a priori, before they enter the panel or a specific sample. This makes the samples vulnerable to fake or bogus respondents who falsely claim to have a profile to meet qualification requirements (for a quota sample [e.g. falsely claiming to be Latino to fill a quota]) (42). Probability samples are less vulnerable on this count because the researcher identifies and approaches respondents from the sampling frame rather than vice versa. Put differently, the recruitment process is well understood in probability samples and is often not in nonprobability samples (43, 44).

A severe case of a bogus respondent is one based on artificial intelligence (AI). While researchers using any sampling approach need to be attuned to AI, the threat seems substantially greater when the AI can opt-into a sample than when a participant is a priori identified (and then potentially uses AI). The ANES and GSS additionally benefit with their face-to-face mode in which bogus or AI concerns become virtually nonexistent. Along these lines, Westwood (45) showed that a relatively basic AI bot passes nearly all commonly used online survey filters and alters survey results (these concerns are distinct from debates about using AI as synthetic respondents). Westwood stated, “the social science community may need to reconsider its heavy reliance on unverified online surveys and reinvest in alternative data collection approaches—such as face-to-face interviews, student samples, administrative records, and other observational datasets—that are more resilient to this form of compromise.” A distinct concern comes from reliance in panels on professional respondents who take many surveys over time and may differ from nonprofessionals (46). Panelists in probability panels tend to take fewer surveys than in opt-in panels (47), presumably reflecting that they are not professionals in the same sense as those who opt-in to nonprobability panels. Regardless, the ANES and GSS avoid the issue by drawing fresh samples.

In sum, nonresponse in probability samples represents a substantial challenge, but it is not a definitive fatal flaw. Nonprobability samples—typically based on panels—offer an alternative, cheaper approach. Yet, they introduce the potential for nonignorable selection biases (who is in the sample?), and in practice, possible skews from bogus respondents and AI. This is not to say that probability samples are “inherently” preferable, as it depends on one's purpose. For instance, nonprobability surveys may be preferred or may be the only conceivable option for hard-to-survey populations, for large samples, for spatially heterogenous samples, and for samples need quickly. Our discussion should not be read as an indictment of nonprobability approaches, but rather it depends on purpose and resources.

When the purpose of a survey is not only to estimate population values or relationships, but also to track them over time, then using a nonprobability sample comes with additional risks when the prior data come from probability samples. For example, if the values of a variable change between two points in time (e.g. average efficacy declines), it would be unclear whether it reflected actual difference or a switch in sampling. Past use of a probability sample does not necessitate its future use, but it constitutes an additional consideration in the context of long-term infrastructure projects. We next provide selected examples of over-time changes that highlight the value of such data: they provide unmatched perspectives on the evolution of key aspects of American society.

## Public opinion trends

The idea of systematically documenting social trends preceded the infrastructure projects. An early example is President Hoover's 1929 committee on Recent Social Trends that produced a report in 1933. The report is an important moment in applied social science (48). Understanding trends matters for gauging the status of social and political institutions. Moreover, over time dynamics play an essential role in understanding individuals’ actions. People generate counterfactuals when assessing the present and anticipating the future (49), and what happened previously serves as a plausible, albeit often imperfect, baseline/status quo (50). Time series data additionally serve a scientific purpose. Trends offer observations from which research questions spring (25), distinct from other work that focuses on particular over time relationships between trends (51, 52).

Studying public opinion trends raises at least 3 questions. First: how to gauge public opinion? In some circumstances, one might have access to administrative data, but these typically do not provide information about the public's thinking. The census provides some data but tends to have limited items (range), particularly concerning politics. The main alternatives, for reasons already discussed, are the ANES and GSS.

Second, whose opinions should be studied? There are many relevant groups such as those who are more or less influential, young or old, of different social backgrounds, and so on. Here, though, as a starting point, we focus on the general population with some discussion of selected subgroups (i.e. partisanship, race, and gender).

Third, what opinions should be documented? Characterization of a society is inevitably difficult and controversial. Given we have an interest in politics, we start with the point that governments largely exist to provide collective goods to citizens to enhance their welfare. We consequently chart individuals’ economic perceptions and health: specifically, economic status and satisfaction, personal health, and happiness. Moreover, the functionality and legitimacy of any democratic government is enhanced when citizens are satisfied with democracy, are not highly affectively polarized (i.e. having high levels of animosity for the opposing party), feel politically efficacious, and have confidence (trust) in societal institutions (to a point) (53). All of these variables also align with the NSF's mission of advancing national health, prosperity, and welfare. Our point is not to offer exhaustive treatment of these metrics or arrive at definitive conclusions about American society and politics. Rather, we seek to demonstrate how informative social trends can be.

To be clear, data from the infrastructure surveys are widely used, and in many ways form the foundation for much of the social sciences. This includes expansive work that employs trends from these data to look at general patterns, variations between and within groups, trends across variables and countries, and comparisons with other data [for reviews, see (3–5, 54)]. Some of this work is in fact formalized in *Public Opinion Quarterly*'s long-standing articles on trends or what are now called polls in context. In a sense, we are reiterating what all of this work demonstrates. Our value added is to make this point in the context of data from both the GSS and ANES and with a focus on individual and national welfare (instead of the common focus on a single topic such as a given public policy). This highlights the value of trends to those who have not yet used them.

## Results

In presenting the trends, we opt for simplicity, leaving it to those who study specific trends to take more sophisticated approaches. For instance, we ignore practices in some specific literatures (e.g. excluding those younger than 25 years of age when looking at happiness) (55). Additionally, in the main text, we merged data across modes of data collection. We used available sample weights that merge across modes. These weights are based on demographic benchmarks and do not include presidential results. Documentation and codebooks for each of the ANES and GSS are available at https://electionstudies.org/data-center/ and https://gss.norc.org/us/en/gss/get-the-data.html, respectively. The ANES introduced an online sample mode in 2012 and the GSS did so in 2021. It is well documented that mode can influence the nature of a sample (56 ) and the responses (57). We thus present results with only the face-to-face mode in the Supplementary material (the general trends remain the same). In the Supplementary material, we also present the trend in affective polarization by mode (given mode has been shown to influence these scores) (58). We display figures with the question wording, N, and data source in the title. We present scores as mean ± SD, reporting proportions in the Supplementary material for scales with 4 or fewer response categories.

### Individual welfare

**Fig. 1.**
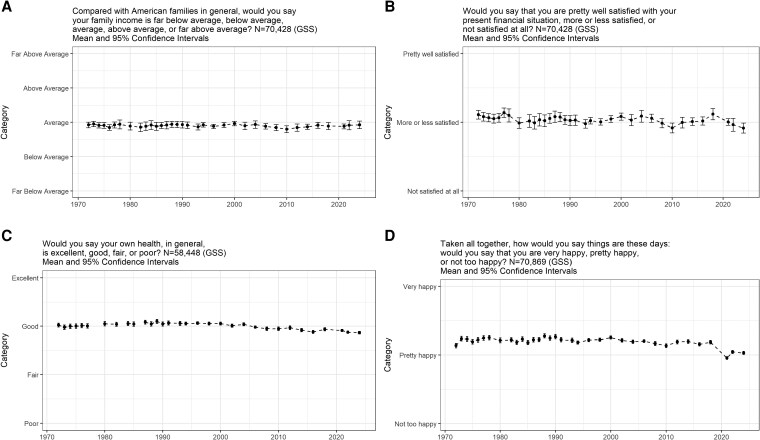
Individual welfare trends. A) economic perceptions. B) satisfaction with financial situation. C) personal health. D) happiness.

We start with individuals’ economic perceptions, perceptions of their personal health, and happiness. Figure 1A presents the average scores of where one perceives their family income to fall, ranging on a 5-point scale from “far below average” to “far above average.” We see virtually no change, with the mean registering around “average” throughout (e.g. the mean scores in 1972 and in 2024 were both 2.92). This should not be taken to mean that individuals are unresponsive to changing economic experiences. This is evident in a distinct item that asks about satisfaction with one's financial situation with the answer options being “not satisfied at all,” “more or less satisfied,” and “pretty well satisfied.” Figure 1B shows a similar underlying stability with the typical score being around “more or less satisfied.” Notable declines occurred after gas prices skyrocketed in the 1970s and early 1980s, with the Great Recession, and with the COVID-19 shutdowns. Satisfaction had not bounced back up by 2024, likely reflecting inflation and high prices. For instance, the 2018 average is 2.12 ± 2.04 (n = 2,314) while the 2024 average registers 1.91 ± 2.11 (n = 3,265), a significant difference (*t*_4988_ = 3.73; *P* < 0.01). The figure suggests reactivity to economic conditions, which itself is a stable reaction to reality. Reactivity though is far from dramatic.

In Figure 1C, we present trends for personal health. We find a slight decline on average by 2024. For instance, in 1972, 2000, and 2024, the average scores on the 4-point scale are 3.02 ± 1.72 (n = 1,612), 3.06 ± 1.83 (n = 2,324), and 2.87 ± 3.07 (n = 3,294), respectively (comparing 2000 to 2024, (*t*_4727_ = 2.90; *P* < 0.01). When we turn to happiness in Figure 1D, we find analogous decline, most notably with the onset of COVID-19 (consistent with increased rates of depression; 59). In 2018, the average happiness score on the 3-point scale is 2.19 ± 1.97 (n =2,344); it dropped to 2.03 ± 2.12 (n =3,281) in 2024, a significant decline (*t*_5064_ = 2.91; *P* < 0.01). The United States is well understood to be modest or low on happiness relative to other advanced industrial nations (60), and the country has ostensibly become recently less happy. Even so, it seems fair to characterize all these shifts as minor. Overall, the individual welfare trends suggest stability but with recent slight downward shifts when it comes to economic satisfaction, health, and happiness. We next turn to opinions about the country.

### National welfare

**Fig. 2.**
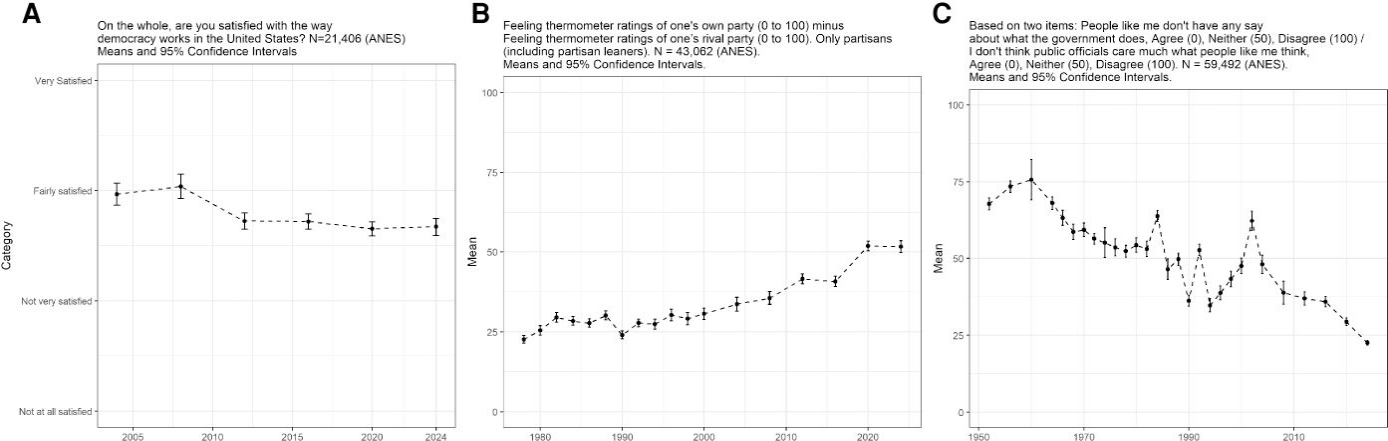
National welfare trends. A) satisfaction with democracy. B) affective polarization. C) external efficacy.

We begin with satisfaction with democracy in Figure 2A (it was first measured in 2004). We find a large decline from 2008 to 2012, with the average moving from 3.04 ± 2.40 (n =1,806) to 2.70 ± 2.63 (n =4,723) (*t*_3998_ = 5.53; *P* < 0.01). We next turn to an item with a longer time series—affective polarization—that captures the difference between partisans “like” for their party and to their “like” for the rival party (each measured on a scale of 0 to 100). For this trend, we only include partisans (including independents who lean toward a party) (61). Figure 2B shows that trend is stark, as is documented elsewhere (62): since the turn of the century, there is near-monotonic increase in affective polarization, with the only exceptions being a slight drop in 2016 and no noticeable change in 2024. In essence, there has been a roughly 30-percentage-point increase in average affective polarization since 2000. Much of this reflects increasing hostility toward the other party, with the average out-party rating going from 41.01 ± 31.29 (n =1,528) in 2000 to 19.27 ± 34.69 (n =5,079) in 2024 (*t*_2467_ = 23.23; *P* < 0.01) (these scores are not explicitly shown in the figure, which depicts the difference between in- and out-party ratings).

The last two trends involve how people perceive their own relationships with the political, economic, and social systems. Figure 2C depicts external efficacy, which gauges agreement or disagreement that people like the respondent have a say in what government does and that public officials care about what people like the respondent think (rescaled to run from 0 to 100, with higher scores being higher efficacy). The figure reveals a steady monotonic decline since an uptick in the 1990s. The 1952, 2000, and 2024 mean scores are 67.82 ± 39.23 (n =1,593), 46.79 ± 46.52 (n =1,354), and 22.12 ± 25.20 (n =4,558), respectively (comparing 2000 with 2024) (*t*_2258_ = 18.716; *P* < 0.01).

**Fig. 3.**
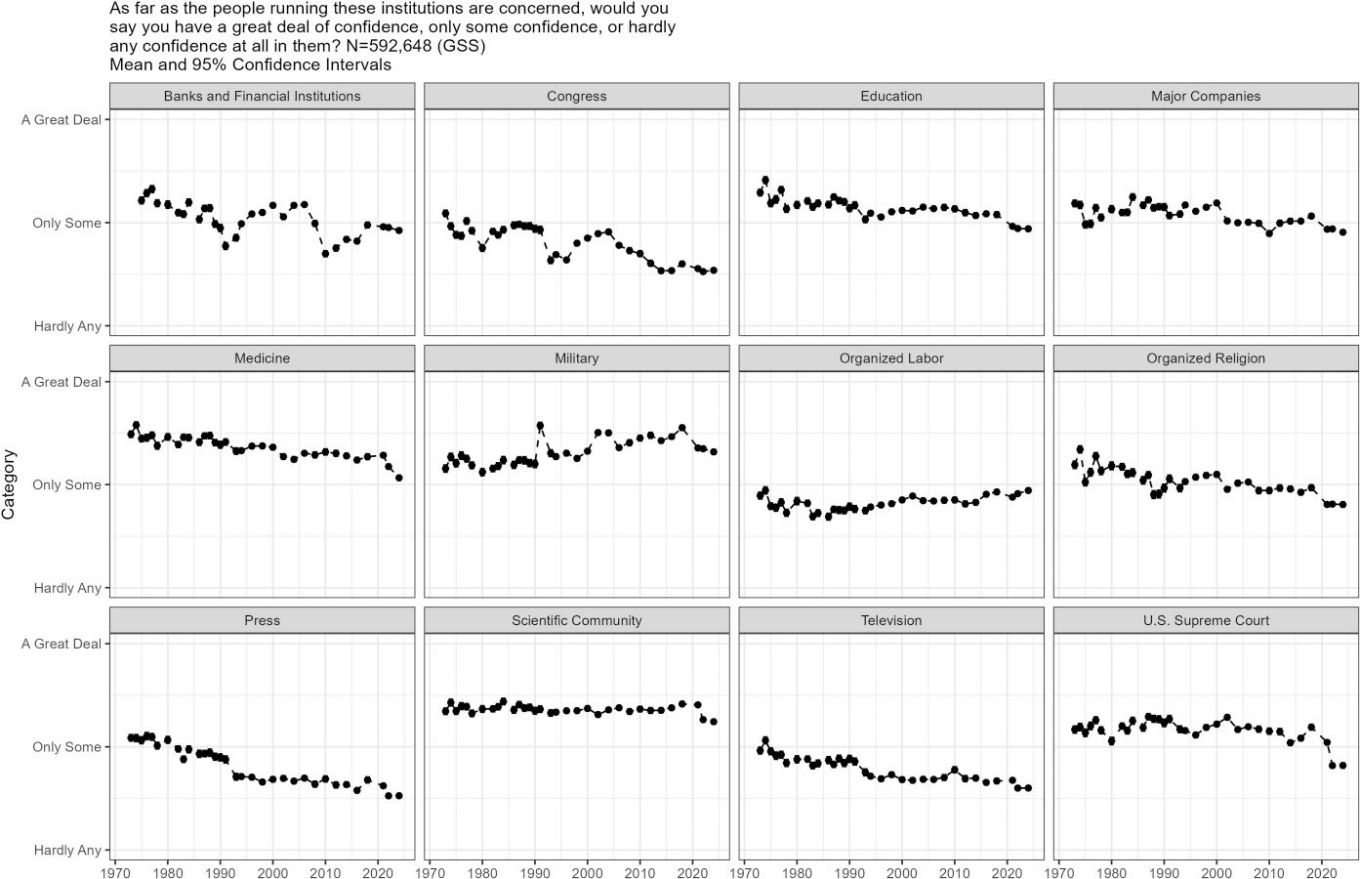
Confidence in institutions.

Finally, Figure 3 displays how much confidence people have in 12 institutions (63)—a metric often treated as akin to institutional trust. [We do not include the executive in the figure, as that largely is a proxy for partisanship, such that trust depends on whichever party controls the presidency (64).] Overall, the picture is one of decline. We see sizeable, over-time drops in confidence in banks and financial institutions, Congress, education, medicine, organized religion, and the press, television, as well as very recent sharp drops in confidence in science and the Supreme Court. Consider that the means for banks and financial institutions, education, the press, medicine, and organized religion in 2000 and 2024 are 2.24 (n = 1,996) and 1.93 (n = 2,703) (*t*_2907_ = 4.80, *P* < 0.01), 2.21 (n = 2,007) and 1.96 (n = 2,708) (*t*_2898_ = 3.81, ; *P* < 0.01), 2.66 (n = 1,982) and 2.40 (n = 2,675) (*t*_2815_ = 3.25; *P* < 0.01), 2.10 (n = 2,000) and 1.75 (n = 2,691) (*t*_2877_ = 5.57; *P* < 0.01), and 2.34 (n = 2,015) and 1.99 (n = 2,700) (*t*_2866_ = 5.29; *P* < 0.01), respectively. Confidence in other institutions (major companies, military, organized labor) either stay mostly flat or marginally increase. The decline in efficacy and confidence (trust) is presumably not surprising given the other national welfare trends, as there is likely a relationship between perceptions of performance (e.g. satisfaction with democracy, polarization) and feeling efficacious and trusting. Across all items, we see a nation with increasingly negative perceptions of politics and society.

### Subgroups

In the Supplementary material, we display each of the figures but with differentiated subgroups by partisanship, race (Black and White), and sex (the GSS historically asked respondents to self-identify as Black, White, or other, and thus more racial categories across datasets are not available). There are a few noteworthy findings. First, there are partisan differences across both the individual and national items. Democrats, relative to Republicans, are historically less financially satisfied, less healthy, and less happy. However, the differences are small and are recently nonexistent. Similarly, Democrats over time have exhibited higher levels of affective polarization and lower external efficacy, but those gaps also no longer exist. The most salient differences are partisan gaps in trust, many of which, emerged fairly recently: Democrats display relatively more confidence in education, medicine, the press, science, and television, while Republicans have relatively more trust in the military, organized religion, and the Supreme Court. These divides contrast with those present at the start of the time series in the 1970s when the only clear partisan divides were on major companies (Republicans had more confidence) and organized labor (Democrats had more confidence). These trends speak to the evolution of cleavages in American politics, from being concentrated along an economic divide to including knowledge-based institutions (63, 65).

Second, given the affinity of Black Americans to the Democratic party (66), it is not surprising that the racial gaps algin with the aforementioned partisan divides. Black individuals historically display lower levels of perceptions of their financial situation, financial satisfaction, health, and happiness. They also were higher in affective polarization and lower in efficacy although those differences do not exist in the most recent data. That said, the racial gaps in institutional trust are much less evident than the partisan ones, with the notable exception of Black Americans having always exhibited less trust in science, which is sensible given a history of scientific omission and abuse (67, 68).

Finally with sex, there are interestingly little differences; even external efficacy does not differ by sex despite that women generally have less confidence in their own understandings of politics (internal efficacy) (40). Of course, this is a surface-level analysis by groups, with the more important point being that these data allow for much more fine-grained analyses than on which we have focused.

## Implications of trends

The negative national welfare trends raise concerns about democratic functionality and legitimacy in the United States. It is thus not surprising that the 21st century in American politics has seen the emergence of right-wing populist politicians, most notably the Tea Party and then President Trump. A citizenry with relatively low efficacy and institutional trust aligns with support for an anti-intuitionalist alternative. Moreover, while research makes clear that there is not a direct relationship between affective polarization and political violence (69, 70), less confidence in and dissatisfaction with democratic institutions could contribute to violence being viewed as an alternative route to settling political disagreements (58). It is thus telling that political violence in the United States has seemingly increased (71, 72). Of course, more work is needed to tie the survey trends to political behaviors (i.e. we are speculating), but the ostensible connections are palatable.

On the flip side, when it comes to explaining the drivers of the survey trends, several candidates exist: the changing demographics of the country that generate threat and democratic dissatisfaction (73), a transformed media environment that can contribute to vilifying opposing sides (74), and growing economic inequality that can undermine efficacy (75, 76). All of these trajectories are presumably self-reinforcing.

Our larger point is that these data provide insight into how citizens have reacted to events and possible consequences. They paint a picture consistent with a declining democracy (77, 78). The relative stability of economic well-being and health highlights a contrast between personal experiences and perceptions of the state of the nation. Many questions remain, questions that may not be asked if not for the institutional commitment to collecting the data.

## Discussion

Our intent is not to provide a definitive treatment of individual or national trends. The data we presented are selected from many others that we could have chosen (although to be clear, we chose these trends prior to fully knowing what all of them would show). We also are cautious in inferring too much as the negative national trends mostly capture one period of time and the “baseline” is somewhat artificial—that is, it could be that some of the declining trends reflect that the second half of the 20th century was unusually salubrious. Additionally, we have barely touched on heterogeneities. In short, much more needs to be done.

Regardless, what we can say with confidence is that these trends are reliable and valid because they come from gold-standard samples that rely on identical or near-identical questions over time. These types of data are impossible to recreate once the trend line stops. To appreciate their value, consider how they have affected 2 research trajectories as examples. One of the more eye-popping trends is affective polarization. Iyengar et al. (79) identified this trend in ANES data, contrasting it with conventional treatments of polarization that focus on ideology or issue positions. Subsequent to that article, affective polarization became one of the most studied concepts in politics, across the world. From 1900 to 2011, a Google Scholar search identified 5 papers with “affective polarization” in the title. From 2011 to October 2025, there were 838 such articles. Another example is work on happiness. While that work has a much longer history and expands well beyond survey data, the availability of the GSS trend and the concomitant trends in associated surveys from the International Social Survey Program have shaped what researchers know about happiness [e.g. (55, 80)]. A Google Scholar search on articles that include the term “happiness trend” yielded 0 results from 1900 to 1971 and 257 results subsequently.

Circling back to the data infrastructure, we recognize that any investment brings with it opportunity costs. With that in mind, it is worth emphasizing that the infrastructure projects themselves are far from static, regularly introducing novel methodological advances, many new questions on the surveys, extensions to the core data collections, and extensive educational tools. Moreover, the NSF's infrastructure program has long supported other projects such as the Cooperative Election Study, the Civic Health and Institutions Project, the Collaborative Multi-racial Post-Election Survey, the Collaborative Midterm Study, Time-sharing Experiments for the Social Sciences, and more. We have offered what may read like a spirted argument on behalf of the infrastructure projects, particularly the ANES and GSS. In the end though, our goal is to explicate a taste of what these projects provide. One could reasonably argue that the resources are better spent collecting data from larger more heterogeneous demographic and/or geographic samples, from more time periods, or from other data sources altogether (indeed, J.N.D. is a co-principal investigator of the Civic Health and Institutions Project, whose mission it is to collect large nonprobability samples frequently and across states). Such arguments need to proceed by articulating the costs and benefits of a given effort relative to the costs and benefits of the existing efforts (while also considering the status quo advantage of extant projects), given one's resources and priorities. We hope that article helps clarify some of what the current investments provide and the sizeable consequences of discontinuing the projects. It should be clear, too, from our previous discussion that any evaluation of investments in surveys needs to account for current state of survey methodology (e.g. nonresponse bias) (see the Supplementary material).

Science is much better positioned to document and explain trends than it was when Hoover launched the committee on Recent Social Trends, nearly 100 years ago. The infrastructure is well-established and has proven its worth. The other side of the equation is effective scientific communication to wide audiences about the product of the investments (81). The projects invest substantially in communication, but it is a difficult task in a constantly changing communication landscape. Regardless, it is the only way for relevant stakeholders to appreciate National Academy of Science's president Marcia McNutt's point that “science is the best—arguably the only—approach humankind has developed to peer into the future, to project the outcomes of various possible decisions” (82).

## Supplementary Material

pgag002_Supplementary_Data

## Data Availability

All data and code necessary to replicate the analyses are available at https://dataverse.harvard.edu/dataset.xhtml?persistentId=doi:10.7910/DVN/LUFLNC.
