# Supplementary material for: Public opinion trends in American society: Lessons from social science infrastructure
Source: PNAS Nexus. 2026 Feb 24;5(2):pgag002. doi: 10.1093/pnasnexus/pgag002 (PMC12930373; doi:10.1093/pnasnexus/pgag002)
Supplement: pgag002_Supplementary_Data [file pgag002_supplementary_data.pdf]

Appendix

1. Face-to-Face Mode
2. Affective Polarization by Mode
3. Proportions
4. Subgroup Analyses
5. Nonresponse Comparison
6. Survey Research Details

Appendix 1. Face-to-Face Mode

In the figures below, we reproduce Figures 1, 2, and 3 from the main text using only respondents who were interviewed face-to-face (excluding web, phone, and zoom modalities). These data thus do not include data collected during the height of the COVID-19 pandemic (when all were on-line samples). The trends remain largely the same.

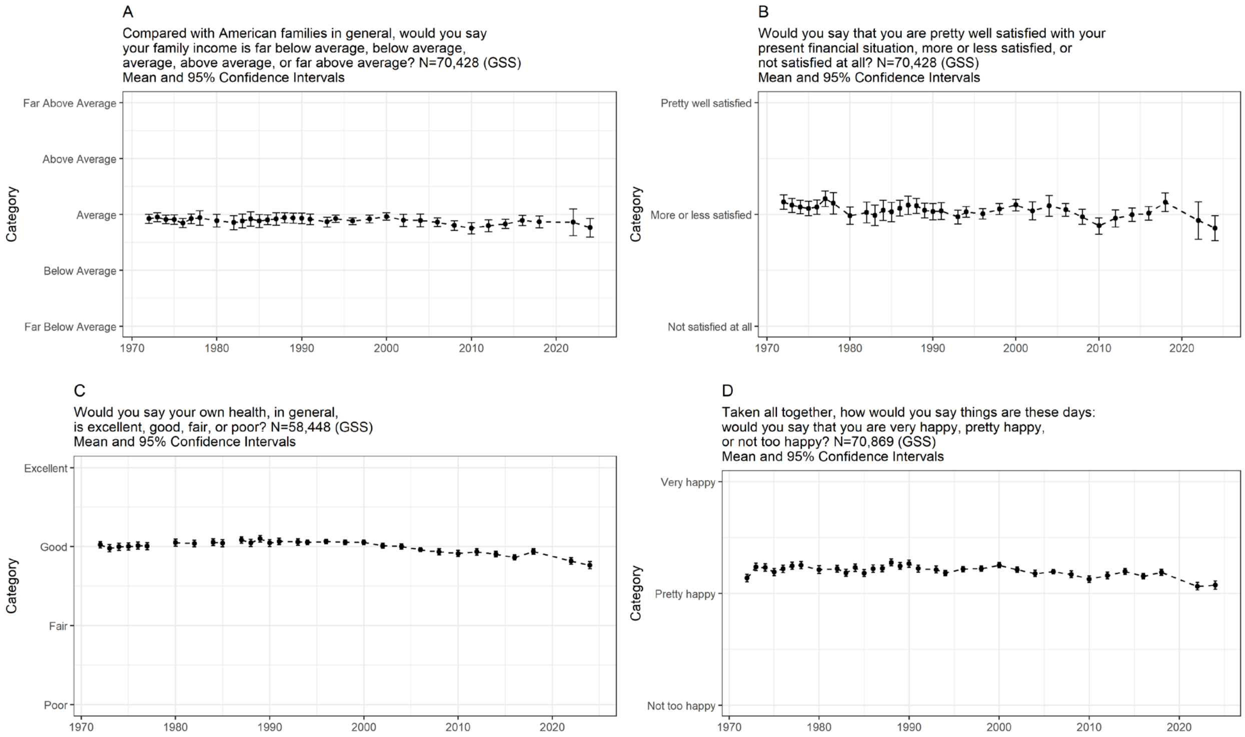

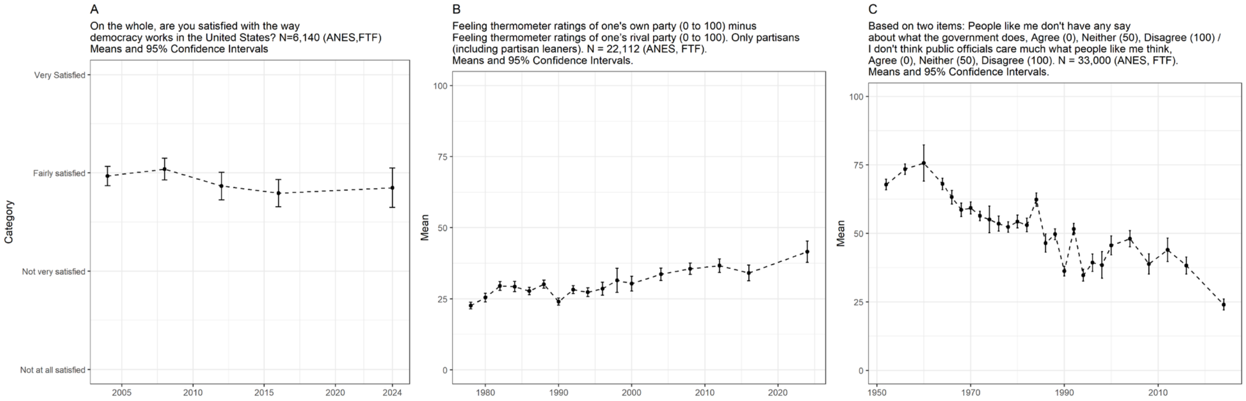

As far as the people running these institutions are concerned, would you say you have a great deal of confidence, only some confidence, or hardly any confidence at all in them? N=513,603 (GSS)
Mean and 95% Confidence Intervals

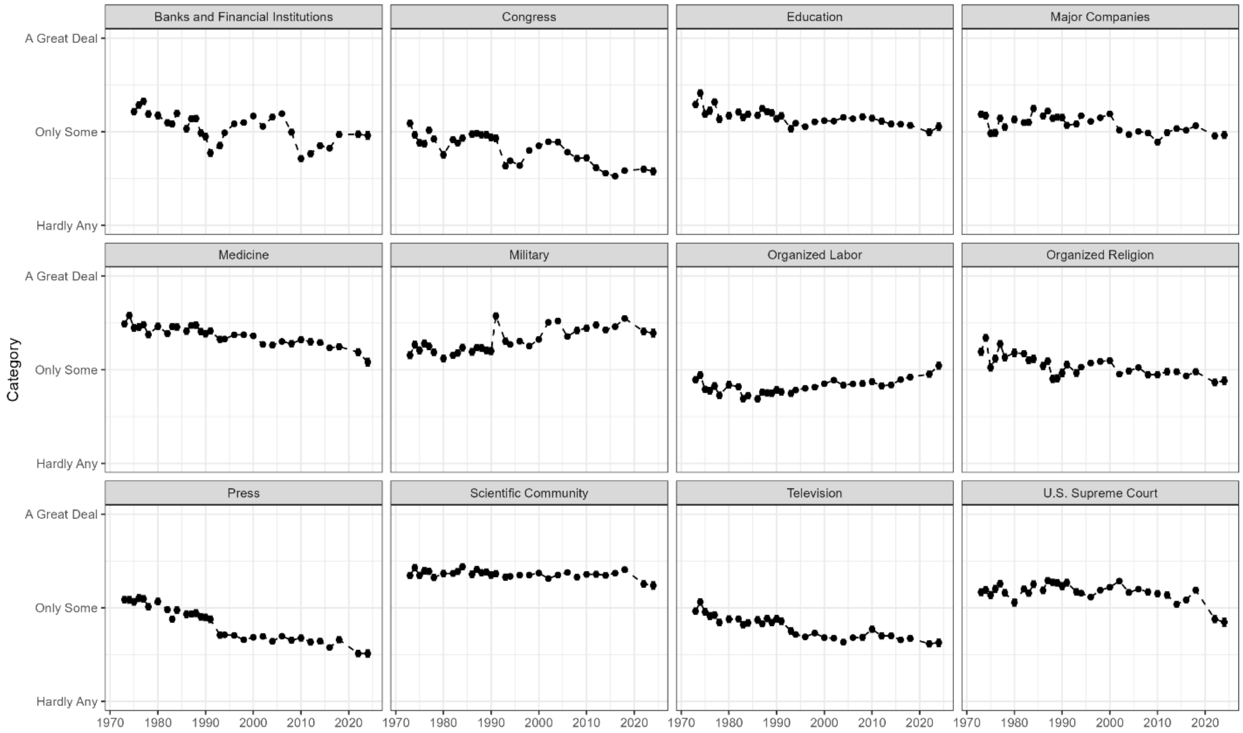

Appendix 2. Affective Polarization by Mode

Affective Polarization is higher among those sampled online relative to those in face-to-face interviews.

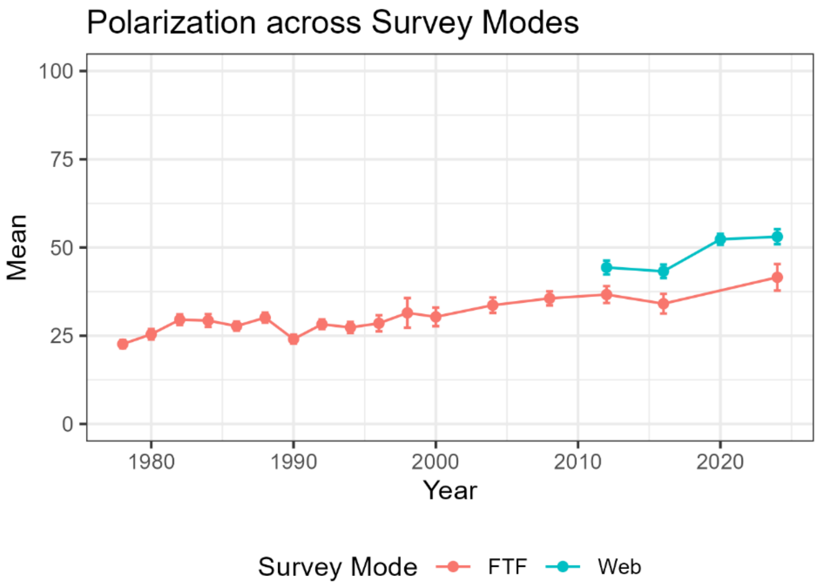

Appendix 3. Proportions

In Figures 1, 2, and 3 in the main text, we plot the average values of a series of variables over time. To do so, we take the means of categorical variables. Here, we show the change in proportions of respondents selecting each category for all variables with four or fewer response options.

Largely, the individual welfare trends are stable over time, with declines in the most positive response categories beginning around 2020. Looking at satisfaction with democracy, there is a slight increase in those who are “Not very satisfied” and a corresponding decline among those who are “Very satisfied” beginning in 2012. Those who are “Not at all satisfied” and “Fairly satisfied” remain largely stable over time. Turning to trust in institutions, the proportion of respondents reporting “Hardly Any Trust” in a variety of institutions (with the exceptions of Organized Labor and Banks and Financial Institutions) increases beginning in 2020.

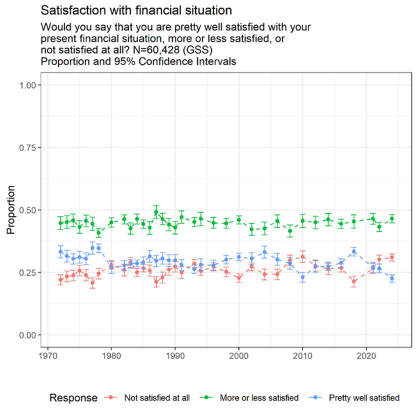

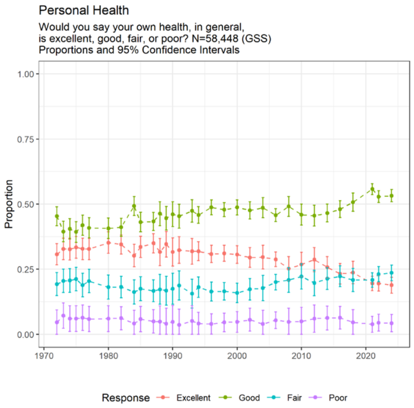

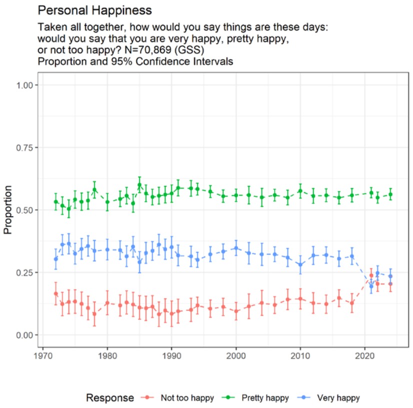

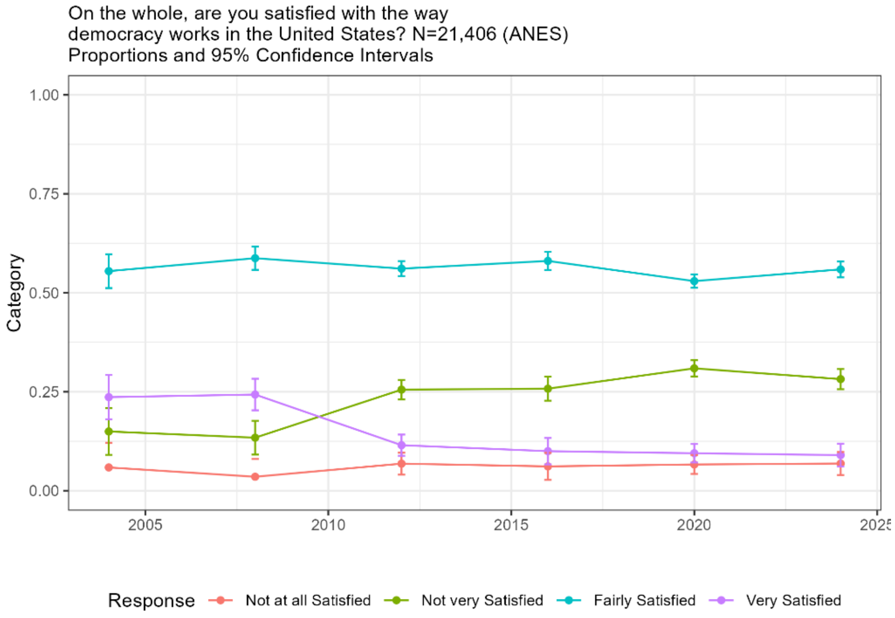

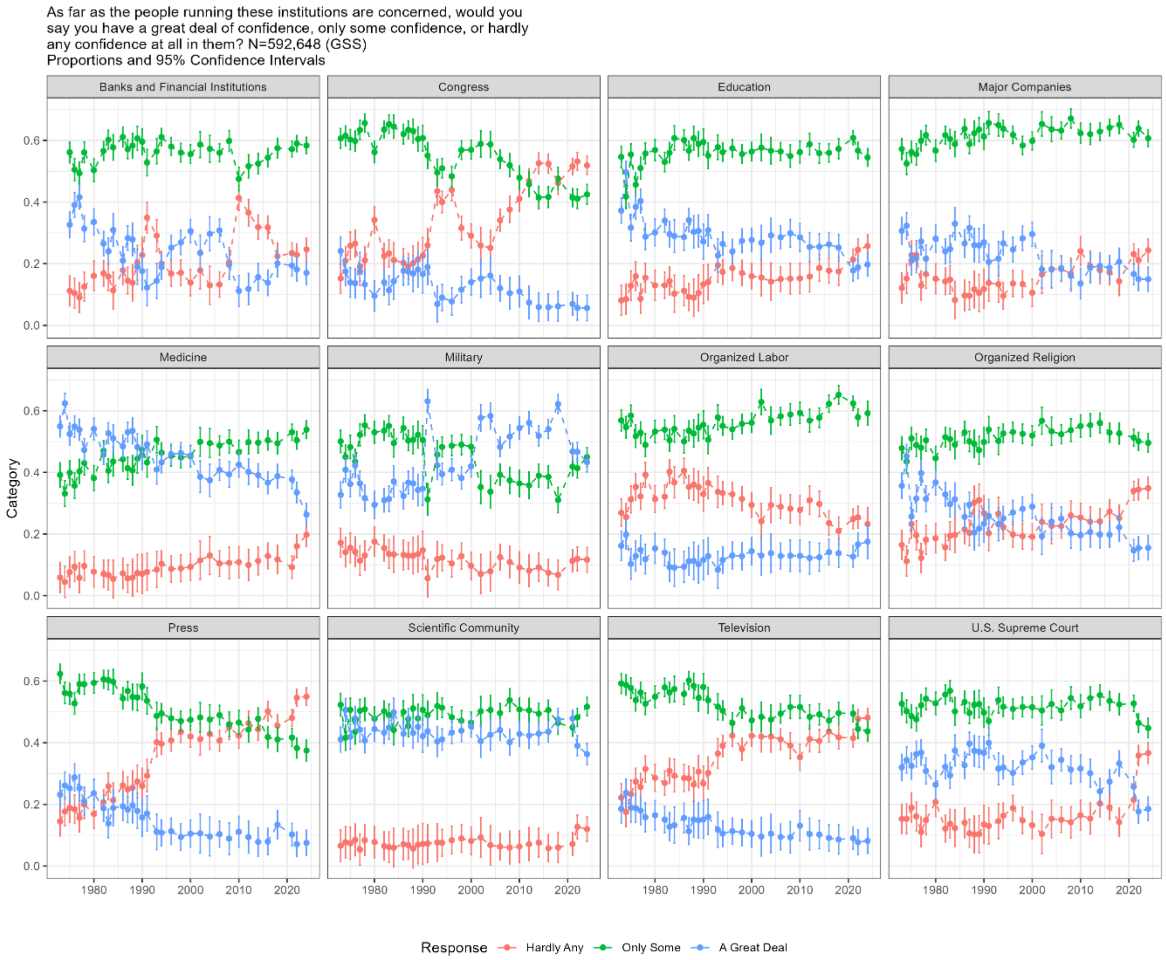
As far as the people running these institutions are concerned, would you say you have a great deal of confidence, only some confidence, or hardly any confidence at all in them? N=592,648 (GSS)
Proportions and 95% Confidence Intervals
Banks and Financial Institutions
Congress
Education
Major Companies
Medicine
Military
Organized Labor
Organized Religion
Press
Scientific Community
Television
U.S. Supreme Court
Category
0.0
0.2
0.4
0.6
1980
1990
2000
2010
2020
Response
Hardly Any
Only Some
A Great Deal

Appendix 4. Subgroup Analyses

We calculate the subgroup means over time for Figures 1, 2, and 3 in the main text by political party, race (white or Black), and sex (male and female).

Panel 1. Individual Welfare

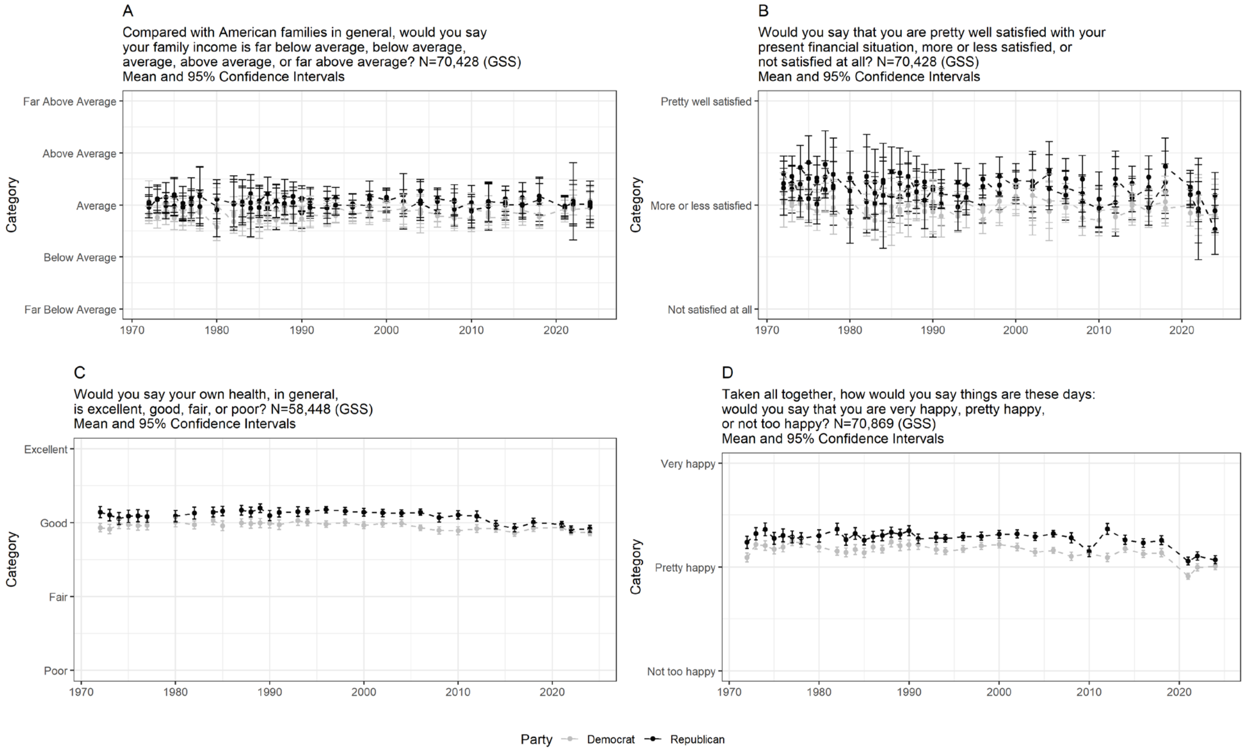

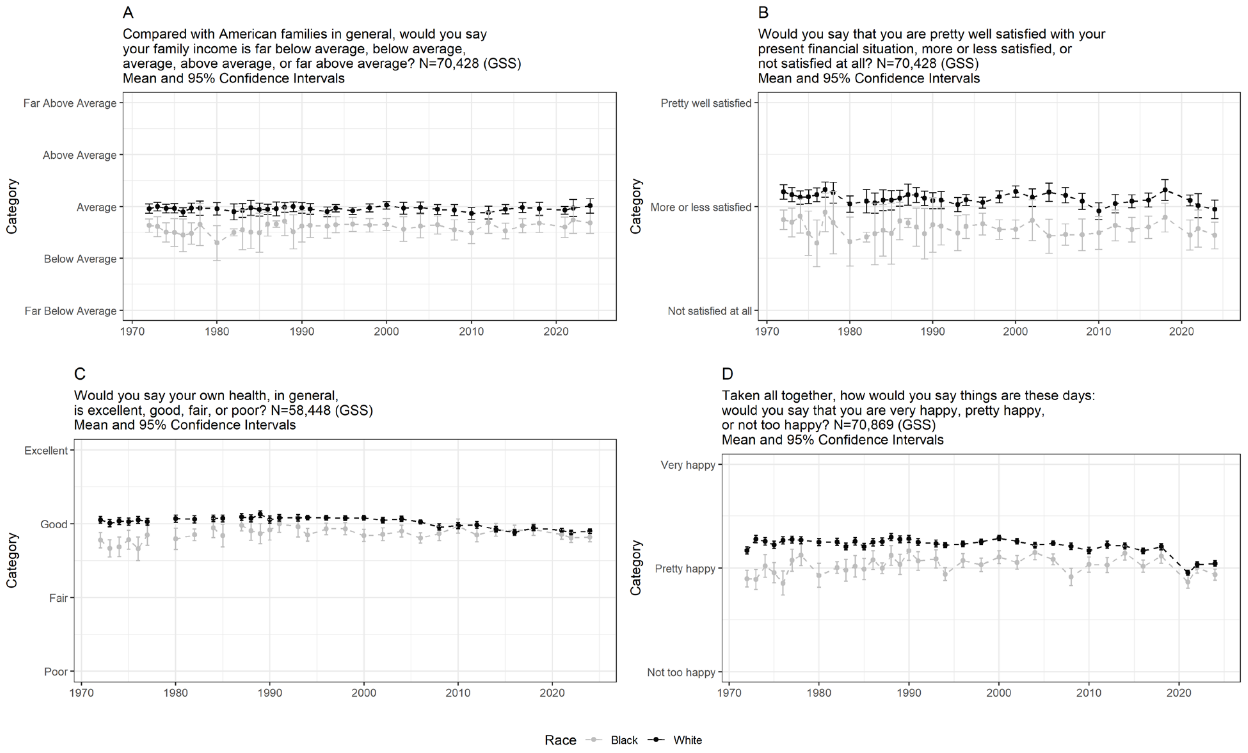
A
Compared with American families in general, would you say
your family income is far below average, below average,
average, above average, or far above average? N=70,428 (GSS)
Mean and 95% Confidence Intervals
Far Above Average
Above Average
Average
Below Average
Far Below Average
Category
1970
1980
1990
2000
2010
2020
B
Would you say that you are pretty well satisfied with your
present financial situation, more or less satisfied, or
not satisfied at all? N=70,428 (GSS)
Mean and 95% Confidence Intervals
Pretty well satisfied
More or less satisfied
Not satisfied at all
C
Would you say your own health, in general,
is excellent, good, fair, or poor? N=58,448 (GSS)
Mean and 95% Confidence Intervals
Excellent
Good
Fair
Poor
D
Taken all together, how would you say things are these days:
would you say that you are very happy, pretty happy,
or not too happy? N=70,869 (GSS)
Mean and 95% Confidence Intervals
Very happy
Pretty happy
Not too happy
Race
Black
White

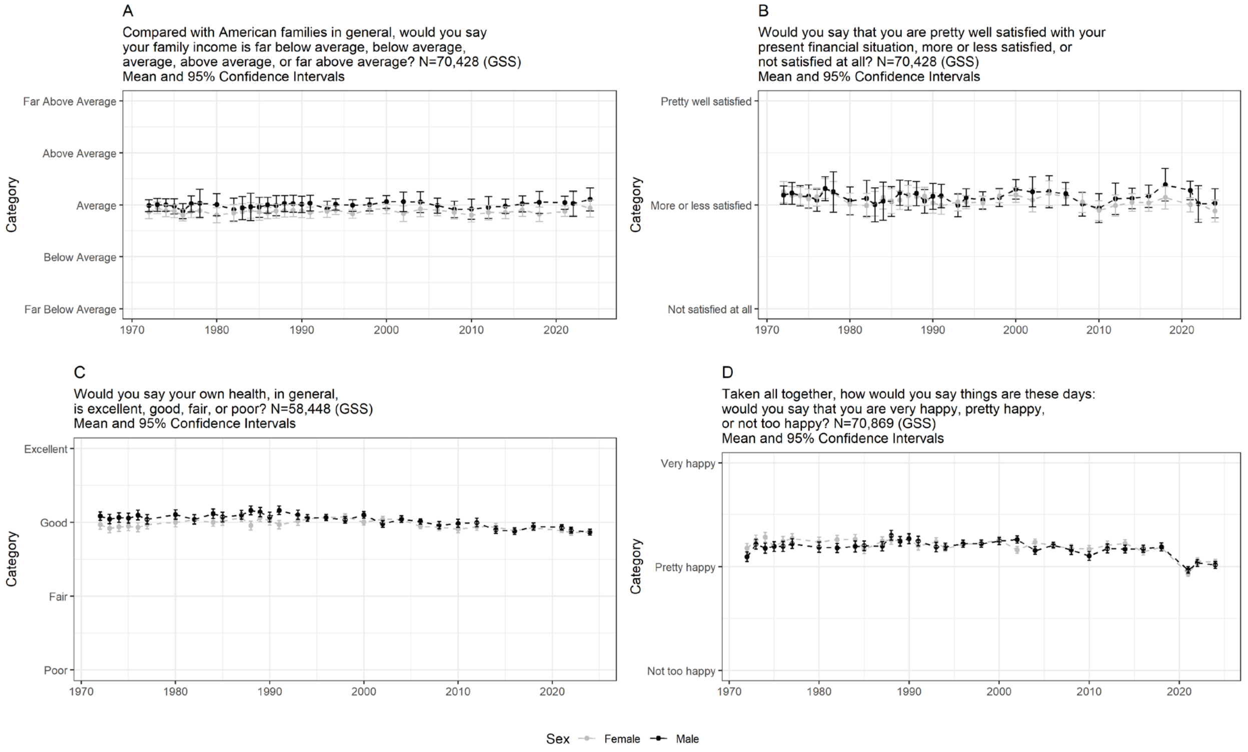
A
Compared with American families in general, would you say
your family income is far below average, below average,
average, above average, or far above average? N=70,428 (GSS)
Mean and 95% Confidence Intervals
Far Above Average
Above Average
Average
Below Average
Far Below Average
Category
1970
1980
1990
2000
2010
2020
B
Would you say that you are pretty well satisfied with your
present financial situation, more or less satisfied, or
not satisfied at all? N=70,428 (GSS)
Mean and 95% Confidence Intervals
Pretty well satisfied
More or less satisfied
Not satisfied at all
C
Would you say your own health, in general,
is excellent, good, fair, or poor? N=58,448 (GSS)
Mean and 95% Confidence Intervals
Excellent
Good
Fair
Poor
D
Taken all together, how would you say things are these days:
would you say that you are very happy, pretty happy,
or not too happy? N=70,869 (GSS)
Mean and 95% Confidence Intervals
Very happy
Pretty happy
Not too happy
Sex
Female
Male

Panel 2. National Welfare

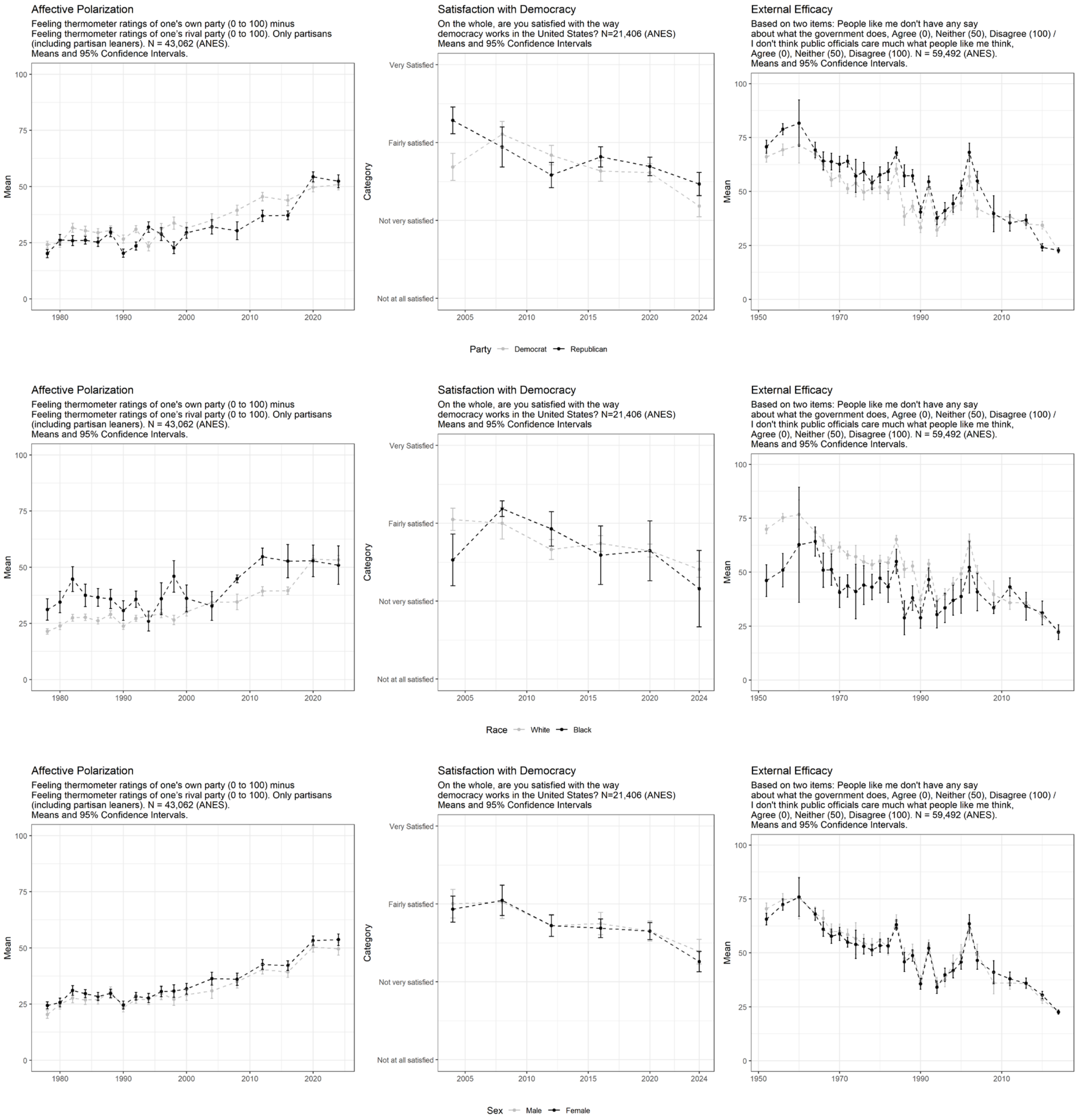

Panel 3. Confidence in Institutions

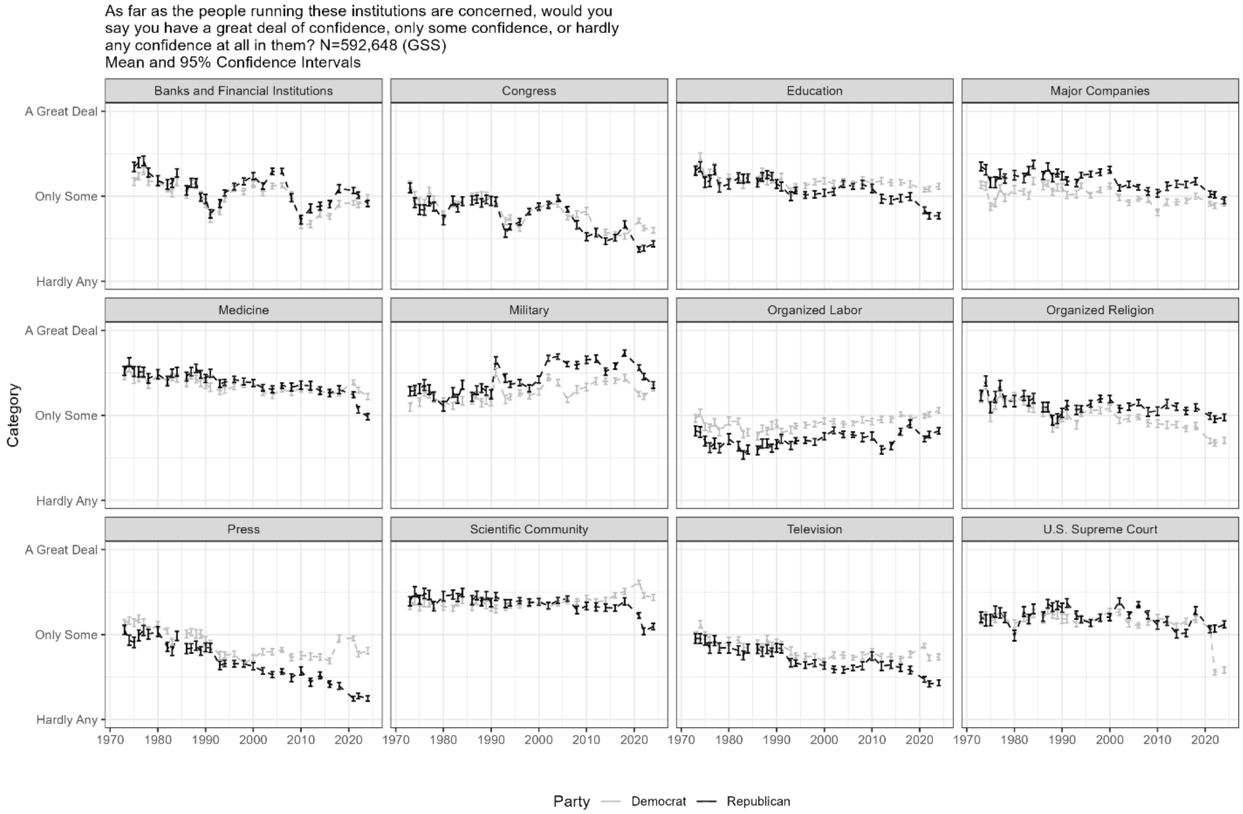

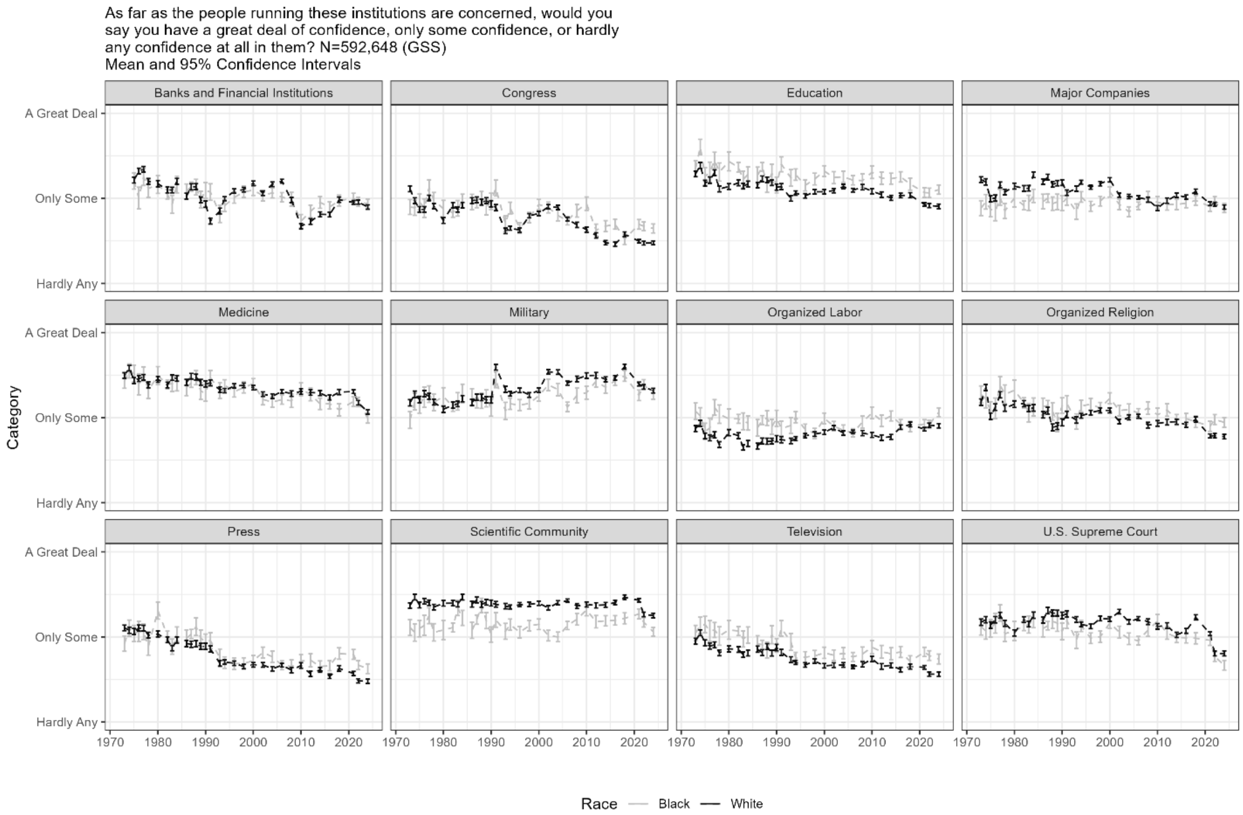
As far as the people running these institutions are concerned, would you say you have a great deal of confidence, only some confidence, or hardly any confidence at all in them? N=592,648 (GSS)
Mean and 95% Confidence Intervals
Banks and Financial Institutions
Congress
Education
Major Companies
Medicine
Military
Organized Labor
Organized Religion
Press
Scientific Community
Television
U.S. Supreme Court
A Great Deal
Only Some
Hardly Any
Category
1970
1980
1990
2000
2010
2020
Race
Black
White

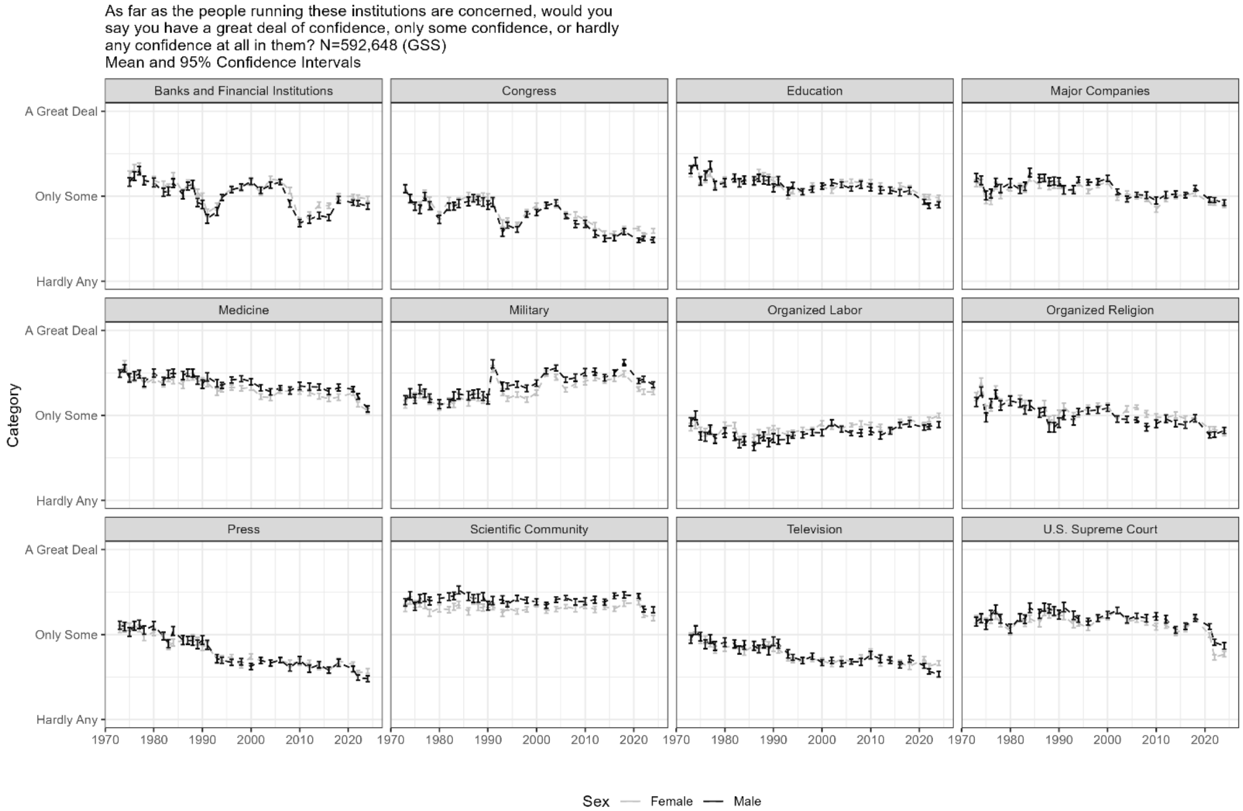
As far as the people running these institutions are concerned, would you say you have a great deal of confidence, only some confidence, or hardly any confidence at all in them? N=592,648 (GSS)
Mean and 95% Confidence Intervals
Banks and Financial Institutions
Congress
Education
Major Companies
Medicine
Military
Organized Labor
Organized Religion
Press
Scientific Community
Television
U.S. Supreme Court
A Great Deal
Only Some
Hardly Any
Category
1970
1980
1990
2000
2010
2020
Sex
Female
Male

Appendix 5. Nonresponse Comparison

In 2020, the ANES attempted to recontact those who did not respond to the original request for contact for the main survey (and thus were non-respondents). It then fielded a brief survey among the non-respondents it reached. Here, we compare the average levels of social trust (one of the few relevant variables between the main and nonresponse study) between the two samples.

The survey asks, “Generally speaking, how often can you trust other people?” and response options range from Always to Never on a 5-pt scale.

Among the 2020 main sample, the mean social trust is 3.19 while it is 3.11 among the 2020 nonresponse sample. These means are statistically distinguishable from each other ($t_{2,556} = 3.29$; $p < .01$).

Appendix 6. Survey Research Details

In all analyses, we used available sample weights that merge across modes. These weights are based on demographic benchmarks and do not include presidential results. Documentation and codebooks for each of the ANES and GSS, respectively, are available at: https://electionstudies.org/data-center/, and https://gss.norc.org/us/en/gss/get-the-data.html. This includes the full question order for each survey over time for both the ANES and the GSS.
